# Transport and Golgi organization 2 deficiency with a prominent elevation of C14:1 during a metabolic crisis: A case report

**DOI:** 10.1002/jmd2.12275

**Published:** 2022-10-27

**Authors:** Katsuyuki Yokoi, Yoko Nakajima, Yoshihisa Takahashi, Takashi Hamajima, Go Tajima, Kazuyoshi Saito, Shunsuke Miyai, Hidehito Inagaki, Tetsushi Yoshikawa, Hiroki Kurahashi, Tetsuya Ito

**Affiliations:** ^1^ Department of Pediatrics Fujita Health University School of Medicine Toyoake Japan; ^2^ Division of Molecular Genetics Institute for Comprehensive Medical Science, Fujita Health University Toyoake Japan; ^3^ Department of Endocrinology and Metabolism Aichi Children's Health and Medical Center Ohbu Japan; ^4^ Division of Neonatal Screening Research Institute, National Center for Child Health and Development Tokyo Japan

**Keywords:** C14:1, eXome Hidden Markov Model, TANGO2, VLCAD, whole‐exome sequencing

## Abstract

Mutations in transport and Golgi organization 2 homolog (*TANGO2*) have recently been described as a cause of an autosomal recessive syndrome characterized by episodes of metabolic crisis associated with rhabdomyolysis, cardiac arrhythmias, and neurodegeneration. Herein, we report a case of a one‐and‐a‐half‐year‐old Japanese girl, born to nonconsanguineous parents, who presented with metabolic crisis characterized by hypoglycemia with hypoketonemia, rhabdomyolysis, lactic acidosis, and prolonged corrected QT interval (QTc) at the age of 6 months. Acylcarnitine analysis during the episode of crisis showed prominent elevation of C14:1, suggesting very‐long‐chain acyl‐CoA dehydrogenase (VLCAD) deficiency. In addition, worsening rhabdomyolysis was observed after intravenous administration of L‐carnitine. VLCAD deficiency was initially suspected; however, the enzyme activity in lymphocytes was only mildly decreased at the gene carrier level, and no mutation in the VLCAD gene (*ADADVL*) was detected. Subsequently, acylcarnitine analysis was nonspecific at 17‐h fasting and almost normal during the stable phase. Eventually, a trio whole‐exome sequencing revealed a compound heterozygous variant of two novel variants in the *TANGO2* gene, a missense variant, and a deletion of exon 7. This is the first case of TANGO2 deficiency in Asians. Our case suggests that elevated C14:1 may be seen in severe metabolic crises and that the use of L‐carnitine should be avoided during metabolic crises.

**Synopsis**

Elevated C14:1 may be seen in severe metabolic crises in TANGO2 deficiency, and the use of L‐carnitine should be avoided during metabolic crises.

## 1 INTRODUCTION

Mutations in transport and Golgi organization 2 homolog (*TANGO2*), which is located on 11.21 of chromosome 22 (22q11.21) and encodes a protein with the same name, were recently described as a cause of an autosomal recessive syndrome characterized by an episode of metabolic crisis associated with rhabdomyolysis, renal complications, cardiac arrhythmias, and neurodegeneration (OMIM 616878).^1^, ^2^

About 90 cases of *TANGO2* bi‐allelic mutation, identified through gene panel or exome sequencing, have been reported in the literature.^3^, ^4^, ^5^

To date, about 30 mutations have been identified in TANGO2 that show an association with TANGO2 deficiency.^4^ The most common variants in patients with TANGO2 deficiency is deletion of exons 3–9 in Caucasian Europeans^6^: however, no clear genotype–phenotype correlations exist.^7^

The age of onset varies from 2 months to 8 years.^5^, ^8^ The severity of TANGO2 deficiency is highly variable. This is not simply due to genotype–phenotype correlations, but it may reflect the accumulation of crisis‐induced damage to tissues, especially in the central nervous system. Metabolic crises are often associated with cardiac conduction defects (most commonly prolonged QTc), which typically normalize between crises.^7^, ^9^

Biochemical findings during metabolic crises include lactic acidosis, hypoglycemia, and mild hyperammonemia.^10^ In addition, the presence of rhabdomyolysis with very high creatine kinase (CK) levels with nonspecific increases in acylcarnitines and dicarboxylic acids are hallmarks of TANGO2 deficiency.^1^, ^10^ However, no specific biomarker of the disease has been identified.^7^

Here, we report the first case of TANGO2 deficiency in Asians with two novel variants.

## 2 CASE REPORT

**FIGURE 1:**
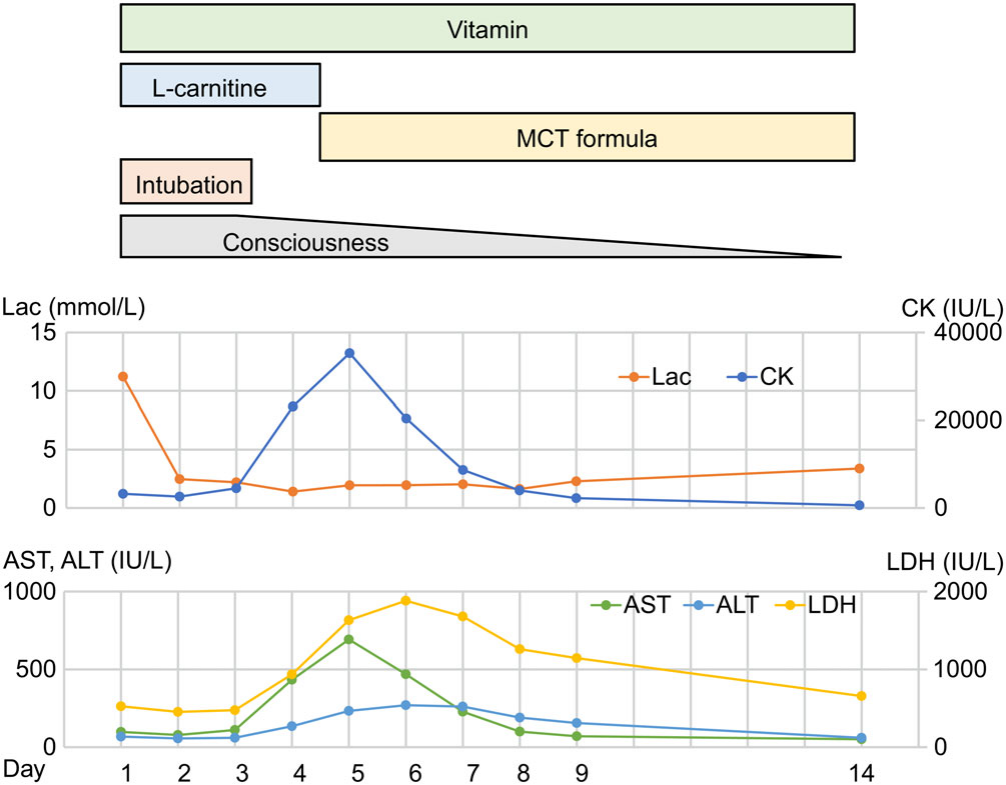
Clinical course during the acute phase along with changes in levels of serum lactate, CK, AST, ALT, and LDH. ALT, alanine aminotransferase; AST, aspartate aminotransferase; CK, creatine kinase; LDH, lactate dehydrogenase; MCT, medium‐chain triglyceride

The patient was a third child with healthy nonconsanguineous parents from Japan. The two older siblings were healthy. The girl was born after a normal pregnancy at 37 weeks of gestation (weight, 2560 g; length, 46 cm; head circumference, 30.5 cm). Postnatal development was initially considered to be normal. Her first episode was at 6 months of age, when she arrived comatose in the emergency room with metabolic acidosis (pH of 7.13) and lactic acidemia of 12.3 mmol/L (normal range: 0.5–1.6). On hospital admission, she was noted to have elevated serum CK level of 3233 IU/L (normal range: 43–165), aspartate aminotransferase (AST) of 98 IU/L (normal range: 8–38), alanine aminotransferase (ALT) of 68 IU/L (normal range: 4–44), lactate dehydrogenase (LDH) of 526 IU/L (normal range: 106–211), ammonia level of 119 IU/L (normal range: 12–66), 3‐hydroxybutyric acid level of 1.7 mol/L, and hypoglycemia (blood glucose 1.8 mmol/L). The remaining liver and kidney function test results were normal. Echocardiography was unremarkable; however, electrocardiogram showed a prolonged QTc interval (593 ms by Bazett formula, normal range <450 ms). Brain magnetic resonance imaging showed no structural abnormalities, but slight nonspecific diffusion‐weighted image hyperintensities in the subcortical white matter and a decrease in the apparent diffusion coefficient value in the same area were observed, suggestive of cellular edema due to metabolic crisis (Data S1). After admission to the intensive care unit, comprehensive treatment including intravenous administration of L‐carnitine, vitamin B1, vitamin B2, biotin, vitamin C, and vitamin B12 were performed. The clinical course during the acute phase and the transition is shown in Figure 1. On the day after admission, her lactate level decreased to 2.4 mmol/L, however, serum CK, AST, ALT, and LDH levels started to rise on the 4th day after admission. The acylcarnitine analysis revealed that several acylcarnitine levels were elevated in both dried blood spots and serum acylcarnitine profiles. Acylcarnitine was analyzed using the non‐derivatized FIA MSMS method. C14:1 was markedly elevated compared to the normal value, suggesting very‐long‐chain acyl‐CoA dehydrogenase (VLCAD) deficiency: dried blood spot C14:1 1.865 μM (normal <0.35), serum C14:1 2.777 (normal <0.2) (Table 1). Therefore, we discontinued the intravenous administration of L‐carnitine and started the medium‐chain triglyceride (MCT) formula. On day 5, CK, AST, ALT, and LDH levels were 35 265, 692, 233, and 1632 IU/L, respectively, and then improved. Similarly, the prolonged QTc interval gradually improved (Data S2). The acylcarnitine profiles were almost normal during the stable phase (Table 1). After her consciousness became normal, a 17‐h fasting test was performed under careful monitoring on day 25 of the illness. The serum acylcarnitine profile showed nonspecific elevations in acylcarnitines: C4OH 0.724, C8 0.421, C10 0.829, C12 0.623, C14 0.347, C14:1 0.877, C16 0.518, C18:1 0.596, C14:1/C2 0.02, C14:1/C16 1.69, and the dried blood spot acylcarnitine profile showed slight nonspecific elevation (Table 1). She was able to walk at 1 year and 5 months, but was still only babbling. She is now one‐and‐a‐half‐year old. No seizures, hearing loss, ophthalmologic abnormalities have been observed yet; however, some biochemical values remained high (CK 533–5195, AST 47–148, ALT 28–186, LDH 301–675 IU/L), and elevated TSH concentration (6.88 μU/mL, normal range: 0.28–4.31) with normal FT3 and FT4 were observed (FT3 4.42 pg/mL, normal range: 2.51–4.16, FT4 1.42 pg/mL, normal range 0.83–1.77). We performed genetic testing of the VLCAD gene (*ADADVL*); however, no mutations were detected. Therefore, we decided to perform whole‐exome sequencing (WES) and search for the cause.

**TABLE 1:** Dried blood spot and serum acylcarnitine profile

Parameter	Stable condition	17‐h fasting test	During metabolic crisis (before loading of L‐carnitine)
Dried blood spot acylcarnitine profile			
C0 (normal >8.00 μM)	52.471	32.537	8.83
C2 (normal <60 μM)	40.282	42.53	20.123
C3 (normal <4.00 μM)	1.949	1.351	1.155
C4OH (normal <0.25 μM)	0.205	**0.467**	**0.266**
C5 (normal <1.00 μM)	0.196	0.111	0.083
C5OH (normal <1.00 μM)	0.71	0.558	0.482
C5:1 (normal <0.025 μM)	0.015	0.011	0.02
C8 (normal <0.3 μM)	0.1	0.229	0.134
C10 (normal <0.4 μM)	0.161	**0.451**	0.37
C12 (normal <0.4 μM)	0.108	0.36	**0.746**
C14 (normal <0.55 μM)	0.189	0.299	**0.771**
C14:1 (normal <0.35 μM)	0.082	**0.471**	**1.865**
C14:1/C2 (normal <0.013)	0.002	0.011	**0.093**
C16 (normal <2.2 μM)	**2.26**	2.157	1.834
C14:1/C16 (normal <0.2)	0.036	**0.218**	**1.107**
C16OH (normal <0.05 μM)	0.024	0.035	**0.062**
C18 (normal <2.2 μM)	1.514	1.097	0.981
C18:1 (normal <4.0 μM)	1.799	1.795	1.296
C18:1OH (normal <0.05 μM)	0.029	0.039	**0.053**

Parameter	Stable condition	17‐h fasting test	During metabolic crisis (after loading of L‐carnitine)
Serum acylcarnitine profile			
C0 (normal range 10–60 μM)	**77.092**	44.135	**116.392**
C2 (normal range 3–30 μM)	21.567	**43.097**	25.599
C3 (normal <2.00 μM)	1.297	0.683	**2.189**
C4OH (normal <0.25 μM)	0.086	**0.724**	**0.305**
C5 (normal <1.00 μM)	0.193	0.118	0.14
C5OH (normal <0.6 μM)	0.06	0.061	0.101
C5:1 (normal <0.025 μM)	0.013	0.013	**0.031**
C8 (normal <0.2 μM)	0.17	**0.421**	**0.251**
C10 (normal <0.35 μM)	0.282	**0.829**	**0.672**
C12 (normal <0.3 μM)	0.142	**0.623**	**1.136**
C14 (normal <0.1 μM)	0.07	**0.347**	**0.932**
C14:1 (normal <0.2 μM)	0.113	**0.877**	**2.777**
C14:1/C2 (normal <0.013)	0.005	**0.02**	**0.108**
C16 (normal <0.15 μM)	**0.175**	**0.518**	**1.137**
C14:1/C16 (normal <1.5)	0.646	**1.69**	**2.44**
C16OH (normal <0.05 μM)	0.012	0.043	**0.09**
C18 (normal <0.15 μM)	0.095	0.134	**0.378**
C18:1 (normal <0.15 μM)	**0.163**	**0.596**	**0.916**
C18:1OH (normal <0.05 μM)	0.008	0.04	**0.059**

*Note:* The value higher than the normal value were bold.

### 2.1 Measurement of VLCAD activity

The enzymatic activity of VLCAD in lymphocytes was measured as previously described.^11^ The result of VLCAD activity performed at day 7 of illness, during rhabdomyolysis, was 35.1% of normal value (48.2 pmol/min/10^6^ cells), which is considered to be at gene carrier level (normal value: 136.4 ± 41.9 pmol/min/10^6^ cells [*n* = 21], gene carrier level: 68.4 ± 23.7 pmol/min/10^6^ cells [*n* = 20]).^11^ The VLCAD activity assay was retested 1 year later during the stable period and found to be in the normal range (patient = 95.4 pmol/min/10^6^ cell).

### 2.2 Genetic analysis

**FIGURE 2:**
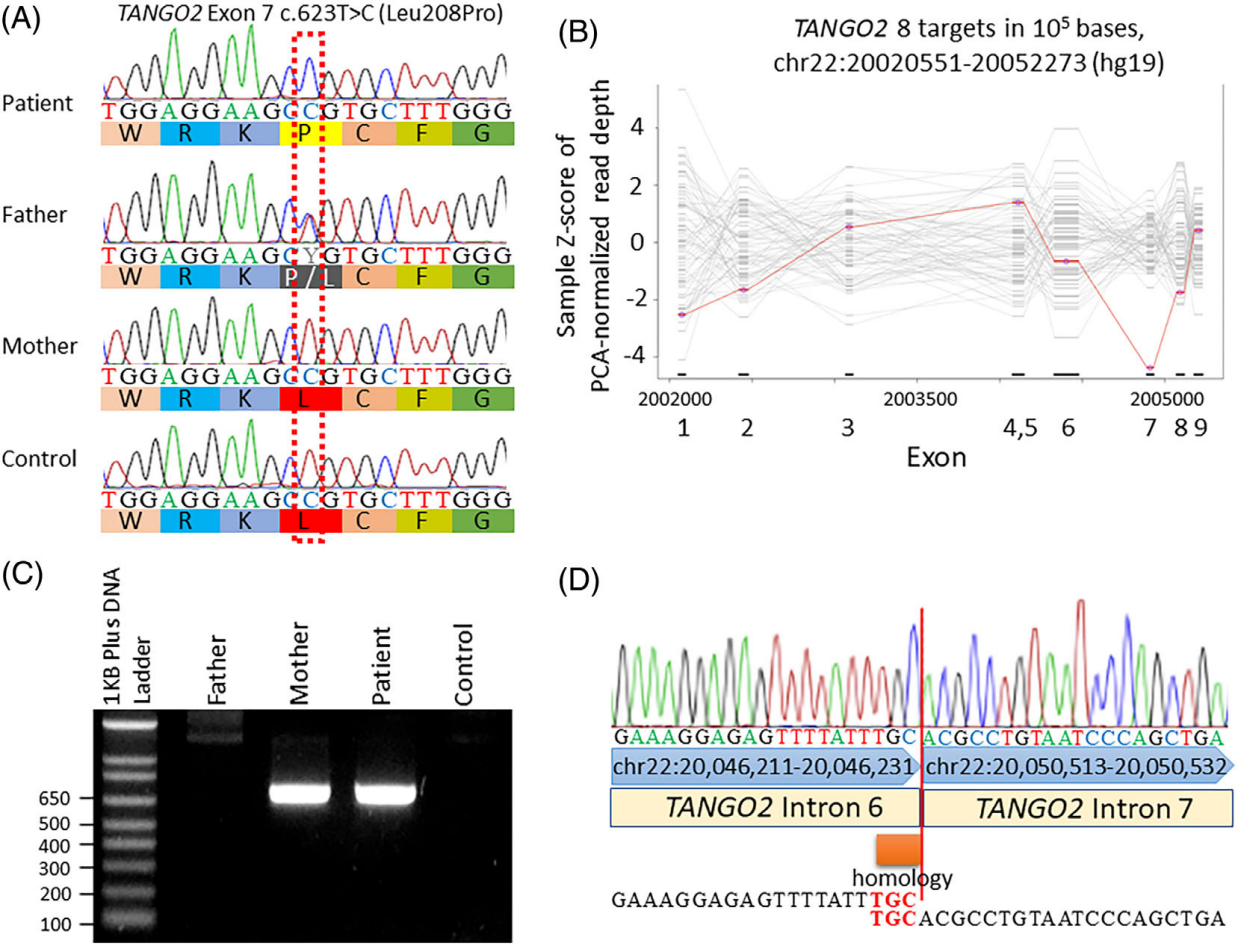
Genetic analysis. (A) Electropherograms of Sanger sequencing results. A homozygous c.623T>C (Leu208Pro) in the patient and heterozygosity in her father are shown. (B) eXome Hidden Markov Model analysis using exome data of 74 samples showed a decrease in read depth in exon 7 at the patient read depth (red line). (C) Agarose gel electrophoresis of the deletion‐specific PCR products. The PCR products were successfully amplified from the patient and her mother. (D) Sanger sequencing of deletion‐specific PCR products, including junctions. The red line represents the breakpoints. The orange bar indicates microhomology at the breakpoint junction

WES was performed using the SureSelect Human All Exon V6 (Agilent) as a probe. Variants with allele frequencies of less than 1% were evaluated. Among these, we selected a missense homozygous mutation, NM_001322141.1:c.623T>C (p.Leu208Pro), on the *TANGO2* gene as a candidate variant. Using direct Sanger sequencing, the variant was confirmed in the father as a heterozygous state, although no variant was found in the mother (Figure 2A). The result suggests that the mother could have some deletion on the allele that was inherited by the patient, or that the patient had paternal uniparental disomy. To determine the copy number abnormality in the *TANGO2* gene, we carried out a quantitative analysis of the patient's exome sequencing data using an eXome Hidden Markov Model (XHMM).^12^ The result indicated that the patient had a deletion of exon 7, in which c.623T>C was located on the other allele (Figure 2B). Deletion‐specific polymerase chain reaction (PCR) confirmed that the deleted allele was inherited from his mother, indicating that the patient was compound heterozygous for the c.623T>C mutation and exon 7 deletion (Figure 2C). Direct Sanger sequencing of the PCR product revealed that the breakpoint junction contained three nucleotides of microhomology at the fusion junction (Figure 2D). Both c.623T>C and exon 7 deletions have not yet been reported. Because the length of exon 7 was 154 bp, the deletion could lead to a frameshift mutation. c.623T>C was evaluated using the American College of Medical Genetics recommendations, and PM2, PM3, PP3, and PP4 were applied, indicating that it is “likely pathogenic.”

## 3 DISCUSSION

In the present case, VLCAD deficiency was initially suspected due to the presence of hypoglycemia with hypoketonemia, rhabdomyolysis, hyperammonemia, and significant elevation of C14:1 in acylcarnitine analysis during the metabolic crisis. Since VLCAD deficiency was ruled out from the enzyme activity test and VLCAD gene (*ADADVL*) analysis, WES was performed, and a diagnosis of TANGO2 deficiency was made. TANGO2 deficiency was first reported in 2016 by two groups.^1^, ^2^ The majority of the reported individuals were of Hispanic or European ancestry. In addition, some consanguineous families from Turkey and of Middle Eastern origin have been described.^10^ Although there have been no reports of TANGO2 deficiency in Asians, it is thought that some cases remain undiagnosed due to difficulty in making a diagnosis and the lack of awareness of the disease.

In TANGO2 deficiency, acylcarnitine analysis was previously reported to be nonspecific; however, there have been some cases with significant elevations of C14:1 during acute episodes.^2^ Similarly, our case showed an increase of C14:1 during metabolic crisis and nonspecific elevations in acylcarnitines in the fasting test, which were almost normal during stable conditions. Furthermore, in the present study, a mild decrease in VLCAD activity (35% of normal value) was observed transiently during the metabolic crisis, which may explain the increase in C14:1 during the crisis. On the other hand, there is a possibility that acylcarnitine results during metabolic crisis were influenced by the combination of fasting and rhabdomyolysis, so the results should be interpreted with much caution. The role of the TANGO2 protein in human metabolism and rhabdomyolysis has yet to be fully examined. A recent study suggested that TANGO2 plays a role in both ER‐to‐Golgi trafficking and in some undetermined processes in mitochondrial physiology; however.^13^ The exact mechanism of the abnormal acylcarnitine levels in TANGO2 deficiency is unclear, but it has been suggested that this is due to impaired fatty acid oxidation caused by a partial reduction in mitochondrial carnitine/acylcarnitine carrier proteins.^8^

In this case, we used lymphocytes to measure VLCAD activity during metabolic crisis; however, to differentiate VLCAD deficiency from metabolic crisis, it might be necessary to measure enzyme activity in a stable state. Our data suggest that the enzymatic analysis using lymphocytes could be directly influenced by metabolic crisis. Furthermore, next‐generation sequencing (NGS) was considered to be useful when biochemical abnormalities normalized between metabolic crises, and differential diagnosis from VLCAD deficiency or fasting was difficult.

It has been suggested that L‐carnitine should not be prescribed for individuals with long‐chain fatty acid disorders during illness, since L‐carnitine may increase the production of long‐chain acylcarnitines and promote toxic effects.^14^ A recent report showed that injection of L‐carnitine during the acute phase increased QT interval in TANGO2 deficiency.^15^ In this case, there was no apparent worsening of the QT interval; however, rhabdomyolysis seemed to worsen during intravenous administration of L‐carnitine and improved after discontinuation which was concomitant with MCT supplementation. These findings also support the idea that long‐chain acylcarnitines are likely to accumulate in patients with TANGO2 deficiency during a metabolic crisis. Since discontinuation of carnitine was concomitant with MCT supplementation in this case, the positive effect of L‐carnitine discontinuation is still unclear. In addition, carnitine deficiency has also been noted in some cases during acute illness, so the need to supplement in certain clinical situations is still an open question.^5^

In this case, exon 7 deletion was detected using the XHMM. Multiplex ligation‐dependent probe amplification (MLPA) and microarrays were used to confirm the copy number variations. However, MLPA is often not available in rare diseases, and microarrays often miss subtle copy number variations. Meanwhile, a recent study has shown that NGS has proven useful for detecting small exon deletions (<10 kb) missed by low‐resolution microarrays.^16^ This report also reiterates the utility of NGS in detecting subtle copy number variations.

## CONFLICT OF INTEREST

The authors declare that they have no conflicts of interest.

## AUTHOR CONTRIBUTIONS

Katsuyuki Yokoi: retrieved the data, and drafted and revised the manuscript. Yoko Nakajima: conception and design, analysis and interpretation, and drafting of the article. Yoshihisa Takahashi and Takashi Hamajima: provided acute phase care for the patient. Go Tajima: measured VLCAD enzyme activity. Kazuyoshi Saito: assessed the cardiac function. Shunsuke Miyai: performed whole‐exome. Hidehito Inagaki: performed the eXome Hidden Markov Model (XHMM). Tetsushi Yoshikawa and Hiroki Kurahashi: experiment analysis and interpretation of data. Tetsuya Ito: conception and design, analysis, interpretation, and critical revision of the manuscript for important intellectual content.

## ETHICAL APPROVAL

All procedures followed were in accordance with the ethical standards of the responsible committee on human experimentation (institutional and national) and with the Helsinki Declaration of 1975, as revised in 2005 (5). The study protocol was approved by the Ethical Review Board for Human Genome Studies at Fujita Health University.

## CONSENT FOR PUBLICATION

Written informed consent for the publication of medical information and images was obtained from the patient's parents reported in this publication.

## Supporting information


**Supplementary Data 1.** Brain MRI during acute metabolic crisis.
No structural abnormalities were found; however, slight nonspecific DWI hyperintensities in the subcortical white matter (a) and a decrease in the ADC value in the same area were observed (b).

**Supplementary Data 2.** Transition of QTcB (in Lead II) during the acute phase (Bazett formula) Each plot represents QTc+/‐SD. The corrected QT interval using the Bazett formula was measured as follows: We selected Lead II and applied the maximum slope intercept method to define the end of the T wave as the intercept between the isoelectric line and the tangent drawn through the maximum downslope of the T wave and measured at least three successive beats; then, we calculated each QTcB, which was defined as QT/ $\sqrt{\mathrm{RR}}$.

## Data Availability

My manuscript has no associated data.
