# Supplementary figures and images for: Transport and Golgi organization 2 deficiency with a prominent elevation of C14:1 during a metabolic crisis: A case report
Source: JIMD Rep. 2022 Oct 27;64(1):3–9. doi: 10.1002/jmd2.12275 (PMC9830013; doi:10.1002/jmd2.12275)

## Slide 1
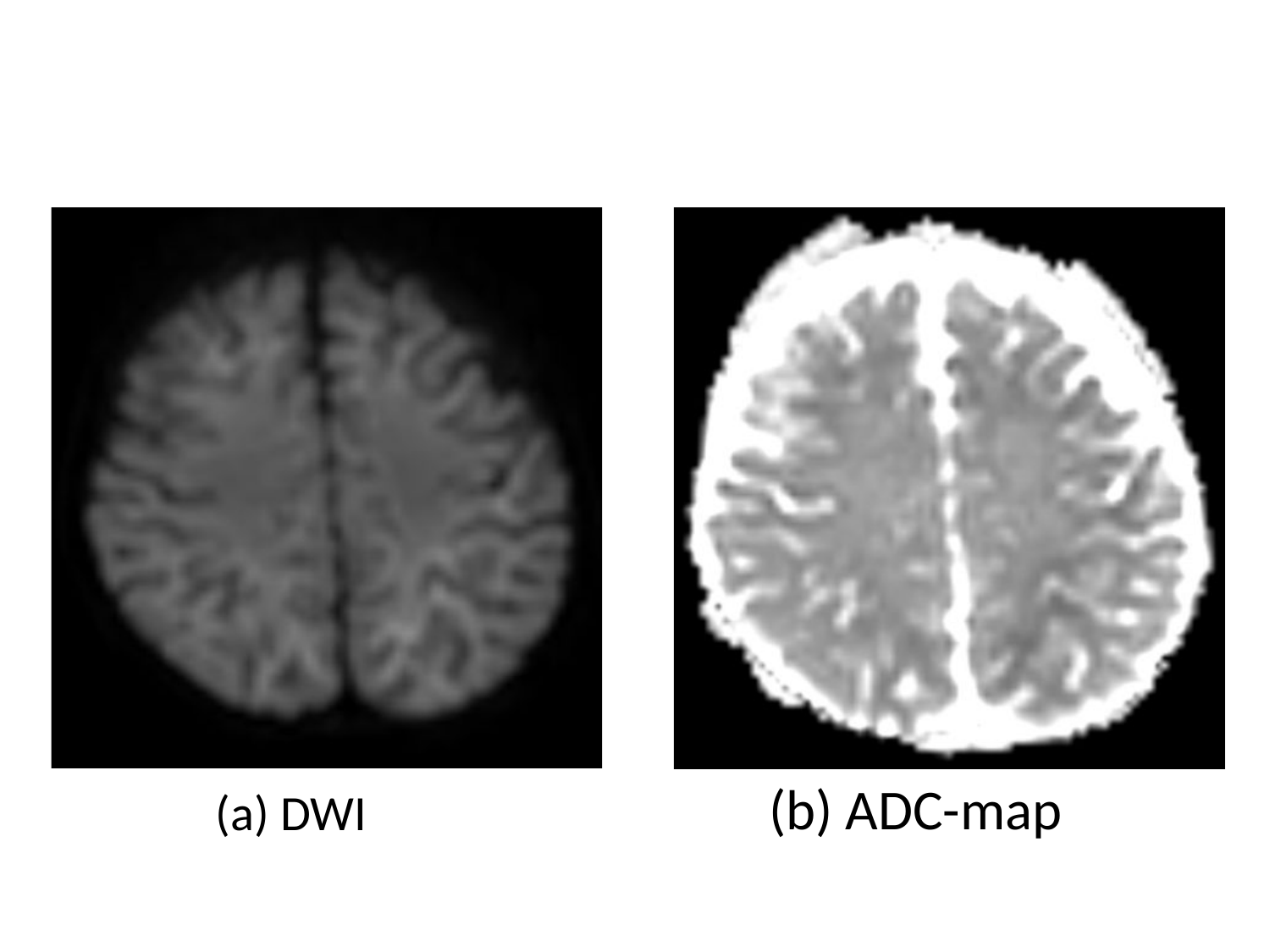

(b) ADC-map
(a) DWI

Supplement: Supplementary file 1 — Supplementary Data 1. Brain MRI during acute metabolic crisis. No structural abnormalities were found; however, slight nonspecific DWI hyperintensities in the subcortical white matter (a) and a decrease in the ADC value in the same area were observed (b). [file JMD2-64-3-s001.pptx]

## Slide 1
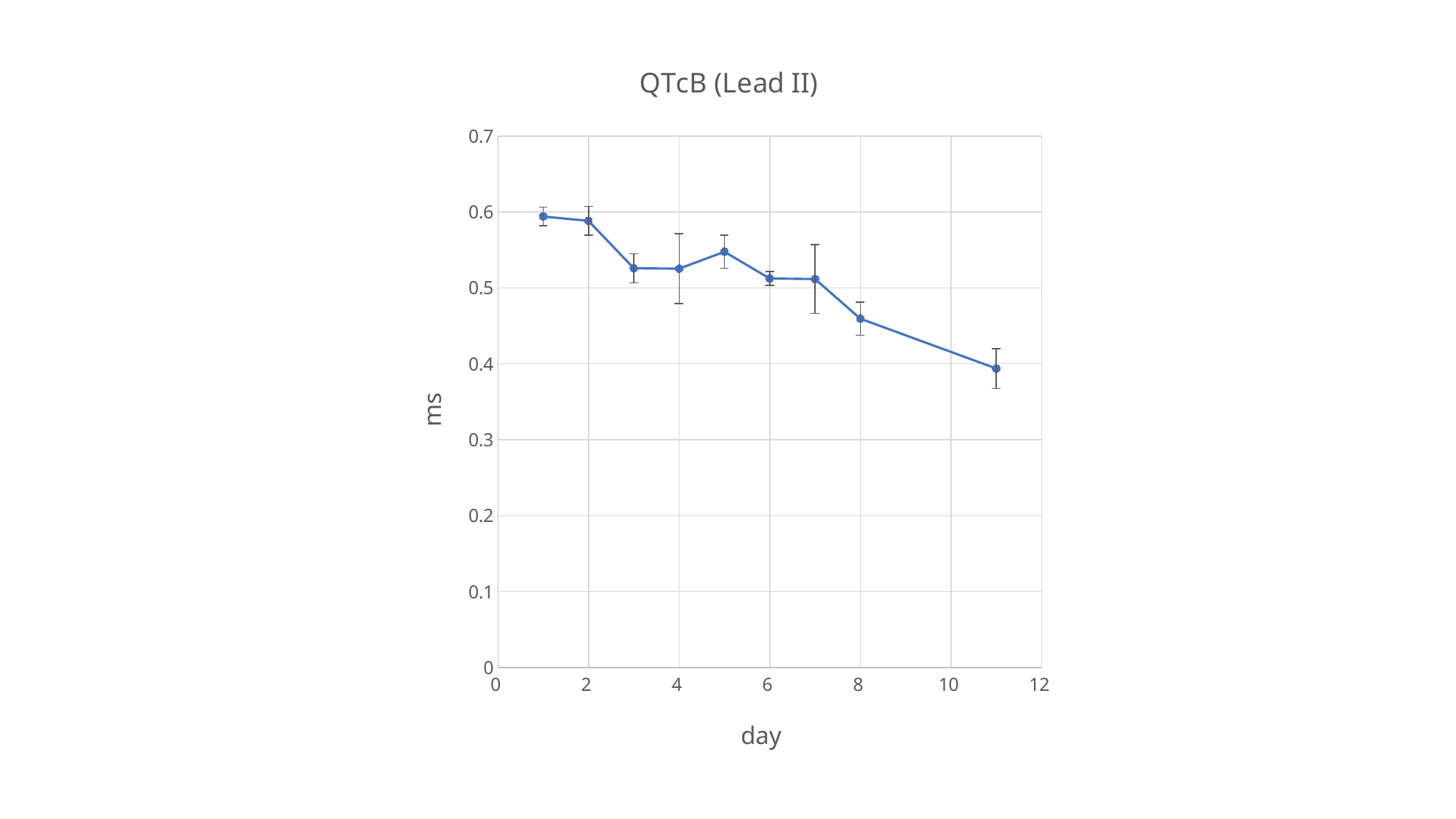

### Chart: QTcB (Lead II)
| Category | QTcB |
|---|---|

Supplement: Supplementary file 2 — Supplementary Data 2. Transition of QTcB (in Lead II) during the acute phase (Bazett formula) Each plot represents QTc+/‐SD. The corrected QT interval using the Bazett formula was measured as follows: We selected Lead II and applied the maximum slope intercept method to define the end of the T wave as the intercept between the isoelectric line and the tangent drawn through the maximum downslope of the T wave and measured at least three successive beats; then, we calculated each QTcB, which was defined as QT/ RR. [file JMD2-64-3-s002.pptx]
